# Breast Cancer Exposomics

**DOI:** 10.3390/life14030402

**Published:** 2024-03-18

**Authors:** Anca-Narcisa Neagu, Taniya Jayaweera, Lilian Corrice, Kaya Johnson, Costel C. Darie

**Affiliations:** 1 Laboratory of Animal Histology, Faculty of Biology, “Alexandru Ioan Cuza” University of Iași, Carol I Bvd. 20A, 700505 Iasi, Romania; 2 Biochemistry & Proteomics Laboratories, Department of Chemistry and Biochemistry, Clarkson University, Potsdam, NY 13699-5810, USA; jayawetm@clarkson.edu (T.J.); corriclg@clarkson.edu (L.C.); johnsokr@clarkson.edu (K.J.)

**Keywords:** breast cancer (BC), exposomics, xenobiotics, breast cancer risk (BCR), biologic pathways

## Abstract

We are exposed to a mixture of environmental man-made and natural xenobiotics. We experience a wide spectrum of environmental exposure in our lifetime, including the effects of xenobiotics on gametogenesis and gametes that undergo fertilization as the starting point of individual development and, moreover, in utero exposure, which can itself cause the first somatic or germline mutation necessary for breast cancer (BC) initiation. Most xenobiotics are metabolized or/and bioaccumulate and biomagnify in our tissues and cells, including breast tissues, so the xenobiotic metabolism plays an important role in BC initiation and progression. Many considerations necessitate a more valuable explanation regarding the molecular mechanisms of action of xenobiotics which act as genotoxic and epigenetic carcinogens. Thus, exposomics and the exposome concept are based on the diversity and range of exposures to physical factors, synthetic chemicals, dietary components, and psychosocial stressors, as well as their associated biologic processes and molecular pathways. Existing evidence for BC risk (BCR) suggests that food-borne chemical carcinogens, air pollution, ionizing radiation, and socioeconomic status are closely related to breast carcinogenesis. The aim of this review was to depict the dynamics and kinetics of several xenobiotics involved in BC development, emphasizing the role of new omics fields related to BC exposomics, such as environmental toxicogenomics, epigenomics and interactomics, metagenomics, nutrigenomics, nutriproteomics, and nutrimiRomics. We are mainly focused on food and nutrition, as well as endocrine-disrupting chemicals (EDCs), involved in BC development. Overall, cell and tissue accumulation and xenobiotic metabolism or biotransformation can lead to modifications in breast tissue composition and breast cell morphology, DNA damage and genomic instability, epimutations, RNA-mediated and extracellular vesicle effects, aberrant blood methylation, stimulation of epithelial–mesenchymal transition (EMT), disruption of cell–cell junctions, reorganization of the actin cytoskeleton, metabolic reprogramming, and overexpression of mesenchymal genes. Moreover, the metabolism of xenobiotics into BC cells impacts almost all known carcinogenic pathways. Conversely, in our food, there are many bioactive compounds with anti-cancer potential, exerting pro-apoptotic roles, inhibiting cell cycle progression and proliferation, migration, invasion, DNA damage, and cell stress conditions. We can conclude that exposomics has a high potential to demonstrate how environmental exposure to xenobiotics acts as a double-edged sword, promoting or suppressing tumorigenesis in BC.

## 1. Introduction

The aim of this review is to deepen our understanding of the study of breast cancer as an ”environmental disease”, using an exposomics-based hypothesis sustaining that BC is an “ecological disorder” [1,2,3]. We are what we eat [4,5,6,7,8], we are what we breathe [9], and we are what we live in [10]. This means that food-borne chemicals, all air, soil, and water pollutants; drugs and drug-related metabolites; different types of radiation; aflatoxins; nanoparticles; noise; and many other environmental factors act, individually or synergistically, as genetic and epigenetic carcinogens, in association with inheritance, disparities, reproductive life, age at exposure, and socioeconomic status, which can also increase BCR [11]. Many studies concluded that cumulative environmental exposure and lifestyle factors account for 70% to 95% of risk factors that drive the BC incidence rate [12], whereas only 10% to 30% of chronic disease risk can be explained by individual genomic landscape [13]. The effects of different types of environmental exposure on BC development, recurrence, overall survival, or treatment resistance [14,15,16] have been reviewed by many authors. Some studies suggest that even climate change will affect women’s cancers [17]. Cell/mobile phone or smartphone use can result in increased BCR, due to the emission of radiofrequency energy that is absorbed by human tissues situated in the proximity, including breast tissue [18]. Many occupational habits, such as heat or night-light exposure, as well as dysregulation of the circadian rhythm, can result in moderate or increased BCR [19,20]. Hair dyes [21], cigarette smoking [22,23], radiofrequency radiation [24], laptops, tablets, and other devices [25], hormone-based treatments [26,27], residential and road traffic noise [28,29], and dust [30] were significantly associated with tumorigenesis and invasive BCR. Last but not least, oncogenic viruses have an important role in BC initiation and development [31].

Exposomics is a modern exposome analysis that characterizes all exposures in an untargeted and comprehensive manner [13]. Thus, the exposome concept is based on the diversity of exposures to physical factors, synthetic chemicals, dietary components, and psychosocial stressors, as well as their associated biological responses [32]. More than two decades ago, Ziegler et al. (1997) reported that BC incidence rates were 4–7 times higher in the United States compared to China and Japan; moreover, when Japanese, Chinese, or Filipino women migrate to the United States, their BCR rates increase over several generations, becoming almost similar with the BCR among American whites [33]. Many studies emphasize that the BCR is elevated compared to countries of origin, mainly due to the exposure to a Western lifestyle [33]. It is known that exposure to a Western diet is a risk factor for the development and maintenance of chronic and systemic tissue inflammation associated with reprogramming of innate immune cells [34]. This lifestyle-associated inflammation is an important cause of multiple cancers, including BC [35]. Recently, the concept of “metaflammation” was used to describe a crosstalk between immune and metabolic pathways that connect obesity to metabolic syndrome (MetS), chronic inflammation, and insulin resistance [36]. It is well-known that MetS is more prevalent in BC patients and is an independent risk factor or predictor for BC [37,38,39].

Consequently, numerous exogenous risk factors influence the growth, proliferation, and differentiation of breast tissue and BC development. A total of 50% of all cancers in women are hormonally mediated, with both estrogen and androgen playing key roles in initiation and BC development [40]. Of all xenobiotic classes, we chose to detail in this review EDCs and food components that can interact with endocrine receptors (ERs) to disturb the normal hormonal equilibrium in BC cells [41]. EDCs can be also ingested with food, so increasing and convincing evidence associates food and food-based dietary patterns with BCR [42]. Moreover, other food components that act as mutagens [43] can be involved in nutritional regulation of the mammary tumor microenvironment (TME) [44], and also impact growth and proliferation of cancer cells [45]. Conversely, food can contain many bioactive compounds with anti-BC potential, exerting a pro-apoptotic role and inhibiting cell cycle progression/cancer cell proliferation, migration, invasion, DNA damage, and cell stress conditions.

It is known that EDC exposure could elevate BCR [46]. Most studies assessed environmental EDC exposure, which includes pesticides, plasticizers, pharmaceutical agents, personal care products, food products, and food packaging, via biomarker measurements [46], so that hundreds of EDCs have been assessed as entering human breast tissue from a wide range of environmental sources, enabling all the hallmarks of cancer to develop in human BC cells [47]. Furthermore, diets comprising energy-dense and nutrient-poor foods have been associated with an increased BCR [48]. Food and food-related/dietary habits, including excessive alcohol use [49], deregulate many signals and metabolic pathways that stimulate the epithelial–mesenchymal transition (EMT), oxidative stress, and reactive oxygen species [50]; dioxin contamination [51], sweetened and highly processed coffee [52] and food [53], meat [54], sweetened drinks [55], EDCs [56], polycyclic aromatic hydrocarbons (PAHs) [57] present in our food, and an inadequate water/liquid daily intake [58] were significantly correlated with carcinogenesis and invasive BC.

Study of absorption, distribution, metabolism/biotransformation, excretion/elimination, and toxicity (ADME-Tox), as well as the bioaccumulation and biomagnification of xenobiotics in cells and liquid or solid tissues emphasize complex interactions with different structures of the human body, such as cellular components (i.e., membranes and proteins), molecular pathways, biological processes, and intra-/extracellular environments [59]. Several exposomics-related omics have been developed as a consequence of advances in molecular sciences and analytical techniques based on high-throughput sequencing and mass spectrometry (MS). Thus, environmental toxicogenomics, epigenomics, and interactomics, metagenomics, nutrigenomics and nutriproteomics, micromiRomics, and nutrimiRomics are several new omics fields related with BC exposomics and are involved in molecular characterization of the complex relationship between the human body, environmental exposure, and breast cancer.

## 2. Advances and Trends in Omics Fields Related to BC Exposomics

Advances in molecular approaches and analytical techniques based on high-throughput sequencing and mass spectrometry (MS) have generated multi-omics data that can be successfully used to understand the underlying molecular mechanisms involved in BC exposomics [60]. BC is mainly caused by mutations in multiple oncogenes and tumor suppressor genes, accompanying epigenetic aberrations of genes and protein pathways [61]. Thus, first of all, environmental toxicogenomics aims to collect, analyze, and interpret data on the changes in genes or protein expression, resulting from exposure to xenobiotics, using high-throughput technologies [62]. Evidence suggests that various pollutants, such as particulate matter involved in air pollution, act as carcinogenic factors in humans, inducing high rates of genomic instability [63], which is known as an initiator of BC development [61]. In addition, environmental epigenomics focuses on environmental factors that induce aberrant DNA methylation of cancer-related genes, even in developing embryos, when result in epigenetic mosaicism that can increase the oncogenic risk later in life [64]. Moreover, metagenomics, the study of genetic information of microorganisms present in an environment [65], is involved in the assessment of the human microbiome as a biomarker that experiences long-term exposure to numerous organic contaminants, known as xenobiotics [66]. Zhang et al. (2028), using liquid chromatography MS-based global metabolomics coupled with targeted metabolomics, demonstrated that the human microbiome can be significantly perturbed by exposure to xenobiotic mixtures, resulting in dysbiosis and metabolite-modified profiles that play an important role in the host’s health [66]. With regard to BC, it is well-known that human microbiome-related disturbance may contribute to BC development by producing toxins or promoting inflammation, while certain types of bacteria may have positive effects against BC [67]. Recently, network biology techniques were used to identify xenobiotics that target hub proteins in the human interactomes, mainly in disease-associated proteins and contaminant-sensitive biomarkers [68], suggesting a new omics field, environmental interactomics. To exemplify, Moslehi et al. (2021) confirmed the role of arsenic as an ED or xenoestrogen involved in breast carcinogenesis, highlighting the complex arsenic-responsive BC interactome [69]. Nutrigenetics studies the effects of nutrition at the gene level, while nutrigenomics is focused on the effects of nutrients on the genome and transcriptome patterns [70]. Thus, based on the complex interaction between food components and human genome/proteome, nutrigenomics and nutriproteomics provide new opportunities for development of personalized diets in patients at risk of developing BC [71].

Tissue or circulating microRNA (miRNA) can serve as a novel toxicological biomarker involved in gene activation or suppression, being associated with several key epigenetic mechanisms involved in xenobiotic toxicity [72,73,74]. miRNAs are also studied and validated as biomarkers for various diseases, as in the case of miR-423, which is highly expressed in BC and promotes cancer cell proliferation, migration, and invasion by activating NF-κB signaling [75,76]. Thus, miRomics is focused on the study of the role of miRNAs in a variety of human diseases, including BC [73]. Evidence suggests that organic pollutant exposure, like bisphenol A (BPA), can alter miRNA expression in response to toxicity [77]. Recently, nutrimiRomics has been defined as a new omics field focused on the influence of diet components on the dysregulation of gene expression due to epigenetic modification that involves miRNAs, resulting in a higher risk for the development of chronic diseases [78]. Thus, Venkatadri et al. (2016) demonstrated that resveratrol, a dietary compound found in a wide variety of plants, can inhibit BC progression by controlling miRNA, regulating the expression of several proteins involved in apoptosis and the cell cycle [79]. These authors emphasized the key role in BC cell death in response to resveratrol for miR-542-3p in MCF7 cell line and miR-122-5p in MDA-MB-231 BC cells [79]. All these new omics fields complement the traditional approach of genomics, proteomics, transcriptomics, and metabolomics, in order to depict the complicated molecular mechanisms studied by BC exposomics.

## 3. Absorption, Distribution, Metabolism/Biotransformation, Bioaccumulation, and Excretion/Bioelimination of Xenobiotics Involved in Breast Cancer

Xenobiotics are substances that are foreign to the intrinsic metabolism of a biological system that has the capacity to bioaccumulate or remove xenobiotics by xenobiotic metabolism, which consists of the deactivation and excretion of xenobiotics and their metabolites [80,81]. The human body is exposed to 1–3 million foreign chemical compounds that form a cocktail/mixture of xenobiotics during a lifetime [82]. In BC, genotoxic carcinogens include dietary or environmental xenobiotics—heterocyclic amines, aromatic amines, PAHs, and nitropolycyclic aromatic hydrocarbons (NPAHs) [83]. Also, many cytotoxic compounds used as anti-cancer drugs for chemotherapy can cause high levels of DNA damage [84], undergo metabolic activation, and are subject to drug metabolism, including uptake, efflux, and detoxification [85].

### 3.1. Absorption

Generally, environmental xenobiotics enter the human body through different absorption surfaces/barriers from input compartments: skin and its appendages, by topical application and absorption, gastro-intestinal mucosa, by ingestion and absorption, and the pulmonary alveolar–capillary membrane, by inhalation. To begin with, EDCs from personal care products are easily absorbed by the skin into systemic circulation after topical application, and can be detected in blood, urine, and breast milk [86,87]. However, Rylander et al. (2019) concluded that intensive use of skin care products did not increase the BCR [86]. On the other hand, 70–100% of patients receiving radiation therapy following BC experienced radiation-induced skin toxicity [88] comparable to UV exposure, which was associated with decreased postmenopausal BCR, due to higher circulating concentration of a precursor to the active form of vitamin D [89]. In addition, the gut absorbs dietary nutrients and provides a barrier to many xenobiotics and microbiome-derived metabolites, so the intestinal epithelium becomes one of the most rapidly proliferating tissues in the body, assuring a rapid and effective elimination of some xenobiotics that bioaccumulate in enterocytes [90]. Consequently, the gastro-intestinal tract is also an important route by which drugs, chemicals, pesticides, environmental pollutants, and metabolites of other species are absorbed in the human body [91]. Last but not least, air pollution is known as a human carcinogen, especially by gaseous components, as well as through particulate matter, including fine, inhalable particles that can be vectors for radioactive isotopes [92,93]. Air polluting agents on their way to the bloodstream pass through the lung barriers [93]. White et al. (2022) showed that higher exposure to ambient particle radioactivity (PR-β) was associated with an elevated risk of ER– BC [92]. Moreover, Smotherman et al. (2023) found a positive association of particulate matter with postmenopausal BCR [94].

### 3.2. Distribution

The distribution compartment, mainly represented by the systemic bloodstream, transports xenobiotics and their metabolites to all tissues and organs, so that blood is the most used liquid biopsy for biomonitoring of xenobiotics, such as persistent organic pollutants (POPs) [95]. From blood, xenobiotics/drugs enter cells, including breast epithelial cells or different cell populations from their ECM or TME. Distribution in cells depend on the chemical nature of xenobiotics, the binding to different receptors or exertion of effects without cellular entry, or using membrane transporters that allow for their entry into the intracellular compartment [85]. Moreover, Ish et al. (2023) showed that changes in breast tissue composition may be a potential pathway by which outdoor air pollution impacts BCR [96]. Thus, quantitative changes in the relative amount of fibro-glandular tissue can represent a biomarker of BCR that can be used to emphasize the potential biologic pathways underlying the association between environmental exposures and BC [96]. In addition, Segovia-Mendoza et al. (2020) showed that the environmental bisphenols, BPA and BBS, induce alteration of the proteomic landscape of different human BC cell lines [97]. After bisphenol exposure, vascular endothelial growth factor (VEGF) secretion, CD44, as a biomarker of stemness, and metalloproteinase MMP-14, as a biomarker for invasion, were overexpressed in ER+ BC cell lines, whereas the epidermal growth factor receptor (EGFR) and transforming growth factor beta (TGF-β) were upregulated in ER– BC lines [97]. Overall, cell and tissue accumulation of xenobiotics, such as EDCs/POPs, could lead to cellular DNA damage and genomic instability [98], epimutations induced by DNA methylation, acetylation, histone posttranslational modifications (PTMs), RNA-mediated effects, and extracellular vesicle effects [99], alteration of DNA methylation during adipocyte differentiation [100] as well as blood methylation [101], epithelial–mesenchymal transition (EMT) by formation of lamellipodia, disruption of cell–cell junctions, E-cadherin downregulation, reorganization of the actin cytoskeleton in stress fibers as well as overexpression of mesenchymal genes, such as vimentin and fibronectin [102,103], FOXA1 repression and phosphorylation of ERK1/2, p48-MAPK, PI3K/AKT signaling in ER-BC cells [104], and upregulation of Snail and Slug in MCF7 ER+ BC cell line [103].

### 3.3. Biotransformation/Metabolism

Many bioreactive compartments are involved in biotransformation and elimination of xenobiotics. Consequently, many chemicals undergo metabolism and detoxification to produce various metabolites that can cause, in turn, harmful effects such as toxicity [105]. Xenobiotic metabolism and detoxification involve xenobiotic-metabolizing enzymes/proteins that are mainly expressed in the liver, but some are also expressed in breast tissue, so that intratumoral xenobiotics or metabolites generated in the liver can undergo further transformation in the breast tissue [83,106]. Thus, many enzymes such as mammary-expressed enzymes metabolically activate or detoxify potential genotoxic BC carcinogens, acting in mammary lipid, nipple aspirate, breast milk, and mammary epithelial cells, where most BCs originate [83]. Bieche et al. (2004) pointed out that the intratumoral dysregulation of genes coding for major xenobiotic-metabolizing enzymes has a role in breast tumorigenesis and drug resistance; thus, N-acetyltransferase 1 (*NAT1)* was proposed as a candidate biomarker for antiestrogen responsiveness [106]. These authors maintained that one-half of the patients with ER+ BC fail to respond favorably to antiestrogen treatment with tamoxifen due to the altered tamoxifen metabolism or bioavailability following the intratumoral alteration in expression of genes coding for xenobiotic-metabolizing enzymes. Moreover, it is known that, in solid tumors, the extracellular and intracellular distribution of xenobiotics and drugs presents a high degree of variability, and is controlled by drug/xenobiotic-metabolizing enzymes (DXMEs) as well as cellular influx and efflux systems that transport xenobiotics and drugs into and out of cells [107].

### 3.4. Bioelimination/Excretion

The main routes of elimination of xenobiotics and their metabolites are renal excretion, bile and fecal elimination, and pulmonary exhalation, but there are also secondary routes, such as sweat, hair and nails, breast milk, and tears [105]. For example, cadmium was detected at high concentration in BC tissue [108], as well as in the urine of patients with BC, urinary Cd being correlated with the expression of hypoxia-inducible factor 1 alpha (HIF1A) in BC tissues [109]. Other heavy metals, such as arsenic, chromium, lead, and mercury are considered to be carcinogens or co-carcinogens and have been detected in the urine of BC patients, even markedly increased [108]. Moreover, the environmental exposure to these heavy metals could influence the urine level of metabolites, in association with BC development [108]. Human breast milk, a specific breast secretion that reflects the molecular landscape of the normal or pathological mammary gland, contains secretions of the mother’s body, in which there are compounds bioaccumulated in her organism, such as organic contaminants (polychlorinated biphenyls, brominated flame retardants, parabens, bisphenols, and perfluoroalkyl and polyfluoroalkyl substances) as well as heavy metals, mycotoxins, and pharmaceuticals residues [110]. Thus, many POPs, such as polychlorinated dibenzo-p-dioxins (PCDDs), polychlorinated dibenzofurans (PCDFs), polychlorinated biphenyls (PCBs), and organochlorine pesticides, such as DDT, have been detected in human blood, adipose tissue, and breast milk and tend to become magnified in the food chain over time; breastfeeding infants becoming the final target of POPs [111]. Moreover, POPs have been correlated to an increased incidence of hormone-dependent BCs [112].

### 3.5. Bioaccumulation

Usually, many xenobiotics, such as POPs and heavy metals, bioaccumulate within adipose tissue, considered to be widely contaminated with lipophilic xenobiotics in modern society and, consequently, acting as a significant site of xenobiotic storage or sequestration [113]. Adipose tissue can play a protective role against xenobiotic effects, because xenobiotic storage in fat can reduce the burden in other critical organs [114]. However, female breast adipose tissue is abundant in and in close contact with epithelial cells, representing a major component of the BC TME, which contributes to the development, growth, and invasion of tumor cells [115]. Weight loss and insulin resistance are involved in xenobiotic release from adipose tissue into bloodstream [114]. Heavy metals, one of the most harmful classes of environmental compounds [116], also stimulate BC progression, exerting a role of DNA methylation level in cancer cells [117]. Heavy metals are very difficult to metabolize or decompose, and accumulate in all tissue and organs over the lifetime [116]. Evidence suggests that obese people accumulate more heavy metals compared to healthy people [118]. Thus, cadmium, among other heavy metals, is a widely spread compound that exerts estrogenic effects, acts as an endocrine disruptor, and accumulates in BC cells over time [109].

## 4. Food and Nutrition

Dietary nutritional intake is a key environmental factor with a vital role in cancer prevention and care [70]. One-third of cancers in Western high-income societies are associated with food and nutrition, in correlation with physical activity [45], so that increasing and convincing evidence associates food-based dietary patterns with BCR [42]. Thus, poor nutrition and foods with a higher energy density have been associated with an increased risk of obesity as well as BC [48,119]. Thus, Jacobs et al. (2021), analyzing dietary patterns correlated to BCR in Black urban South African women, concluded that both traditional and cereal–dairy-based meals may reduce the BCR in this population [48].

Overall, thirteen cancers, including BC, have been estimated to be associated with obesity and are known as “obesity-associated cancers” [120]. The female breast is rich in adipose tissue [121], so that, in postmenopausal women, the adipose tissue becomes a significant source of estrogen, this obesity-associated estrogen likely playing an essential role in BC growth, mainly in ER+ BC tumors [120]. Conversely, caloric restriction or intermittent fasting, a period of voluntary abstention from all food or specific food products [122], can negatively impact BC development, reduce the treatment-induced adverse effects, cytotoxicity, and DNA damage, and increase optimal glycemic regulation, improving serum glucose, insulin, and insulin-like growth factor 1 (IGF-1) levels [123]. Insulin and the IGF-1 pathway regulate lifespan and longevity [124]. IGF-1 is known as a potent mitogen of high importance in the mammary gland that binds to the cognate receptor, IGF-1R, triggering a signaling intracellular cascade, which increases the proliferative and anti-apoptotic pathways in cancer cells [125]. It is known that the Western diet, characterized by high intake of hyperglycemic carbohydrates and insulinotropic dairy, stimulates IGF-1 signaling [124]. GH, IGF-1, and insulin have BC-promoting actions, due to increased IGF-1 levels, which have been associated with increased BCR [124].

Food components may act as mutagens, such as N-nitroso-derivatives, polycyclic aromatic hydrocarbons (PAHs), and heterocyclic aromatic amines [43], which can be involved in nutritional regulation of the mammary tumor microenvironment (TME) [44], and impact the growth and proliferation of cancer cells [45]. Nutritional stimuli modulate interactions between different cell populations within the TME, such as immune cells, adipocytes, vascular cells, and mammary epithelial and BC stem cells, so that both obesity, a chronic over-nutritional condition, as well as excess caloric consumption, disrupt mammary gland homeostasis and increase BCR [44,123]. EDCs has been reported in aquatic macroinvertebrates, mussels and seawater or freshwater fish [126], pork, beef, and chicken meat [127], vegetables [128], as well as in milk and dairy products [129]. Heavy metals, such as cadmium, mercury, and lead, act as EDCs and bioaccumulate mainly in fish and seafood products [130]. Fish product consumption acts as a double-edged sword. There are studies that emphasize the protective effect of omega-3 fatty acid in fish consumption against BC [131], while the human exposure to fat from milk, eggs, fish, and meat can enhance mammary gland susceptibility to carcinogenesis [132]. Alcohol consumption has been related to higher BCR, principally for estrogen receptor-positive (ER+) BCs [133], through stimulation of migration and invasion of MCF7 human BC cells [133], EMT, angiogenesis, OS and ROS production [49,50], decreasing E-cadherin, α, β, and γ catenin expression, as well as *BRCA1* tumor suppressor gene expression [133].

Fortunately, in our food, there are many bioactive compounds that are able to exert an anti-cancer potential, re-inducing apoptosis or targeting multiple signaling pathways that allow for cancer cell survival, proliferation, growth, and metastatic progression of BC cells [134]. Many dietary compounds are also considered epigenetic modulating agents in cancer [135]. Thus, both green or black tea, as well as green or dark coffee, have been associated with a reduced BCR [136,137]. Chlorogenic acid (CGA) from coffee exerts an inhibitory role on signaling pathways, such as NF-κB/EMT [138]. Epigallocatchin-3-gallate from green tea significantly reduces BCR by decreasing ROS and oxidative DNA damage, mutagenesis, and tumor progression [137]. Resveratrol from grapes, berries, and nuts can reduce specific cancer stem cell (CSC) biomarkers in BC cells [139]. Piperine inhibits the growth of human BC cells, cell cycle progression, and BC cell migration [140]. Carotenoids have been associated with several metabolites involved in membrane signaling, immune regulation, redox balance, and epigenetic regulation [141]. One of the most active components of garlic (*Allium sativum*), allicin (diallylthiosulfinate), induces cell cycle arrest and has pro-apoptotic effects in BC cells, through p53 pathway activation [142], exerting antiproliferative, anticlonogenic, and senolytic effects, inducing the selective death of senescent cells [143]. Last but not least, the omega-3 polyunsaturated fatty acids, eicosapentaenoic acid (EPA) and docosahexaenoic acid (DHA), decreased tumor cell proliferation by downregulation of proliferation-associated protein expression (proliferating cell nuclear antigen (PCNA) and proliferation-related kinase (PRK), induced apoptosis by increasing caspase activity and DNA fragmentation, and decreased signal transduction through the Akt/NF-κB cell survival pathway [144].

## 5. Exposure to Endocrine-Disrupting Chemicals (EDCs)

EDCs are man-made chemicals ubiquitously found in the atmosphere as aerosols and particulate matter [145], water [146], pesticides [147], metals such as cadmium (Cd), mercury (Hg), arsenic (As), lead (Pb), manganese (Mn), and zinc (Zn) [148], additives or contaminated food such as dairy products, fish, meat, eggs, and vegetables, bottled water and canned food [149], and cosmetics and personal care products [150]. EDCs arrive in the human body through ingestion, inhalation, and/or the transdermal route, bioaccumulate, and interfere with endocrine, immune, and other systems, leading to a disruption of the endocrine signaling and metabolic pathways, and inducing life-long effects and negative consequences even for the next generation [151]. EDC exposure also interferes with placental function [152], can interfere with gamete quality, embryo implantation, and fetal development, with serious consequences for offspring viability and health [153]. EDCs affect epigenetic markers such as DNA methylation and histone posttranslational modifications (PTMs) [154]. In addition, EDCs increase incidence of BC [151].

EDCs are heterogeneous natural or synthetic compounds that include pharmaceutical agents (diethylstilbestrol (DES)), fungicides and pesticides (dichlorodiphenyltrichloroethane (DDT)), plastics (bisphenol A (BPA)), plasticizers (phthalates), and industrial solvents/lubricants (polychlorinated biphenyls (PCBs), polybrominated biphenyls (PBBs), and dioxins). Many EDCs are persistent organic pollutants (POPs), known as lipophilic toxicants that persist in the environment due to their resistance to biodegradation and, moreover, biomagnify or move up the food webs and increase in concentration [113]. POPs affect the production of estrogens and estrogenic signals, so that, measured in breast adipose tissue, POP levels were associated with higher BCR and worse prognosis [112].

Several pathogenic effects of EDC exposure are presented in Table 1. Thus, BPA stimulates the proliferation and malignancy of cancer cells through the activation of the Wnt/β-catenin pathway [155], which is widely implicated in the pathogenesis of metastatic BC [156]. Significant deregulated gene expression and transcriptional reprogramming in adult fibroblasts exposed to in utero BPA and DES, and specifically, changes in extracellular matrix (ECM) composition due to increased collagen deposition in adult mammary glands, lead to molecular alterations, which develop over time and contribute to increased BCR in adulthood [157]. Consequently, in utero exposure of the embryo to high maternal synthetic estrogens/EDCs could be associated with an increased BCR later in life [158]. Thus, BC may start in the womb, EDCs affecting the early development of mammary glands [159,160].

It is known that African Americans (AAs) are disproportionately exposed to elevated levels of BPA, so that the urinary BPA level among Black adults and children are statistically significantly higher compared to the non-Black population [161]. Recently, Zhang et al. (2023) used a metabolomics-based approach based on both ultra-performance and high-performance liquid chromatography tandem mass spectrometry (UPLC/HPLC-MS/MS) and demonstrated a high connection between tetrabromobisphenol A (TBBPA), a brominated derivative of bisphenol A (BPA) that is extensively present in the environment, with BC development [115]. In male and female rats and Rhesus monkey, low-dose exposure to BPA can affect mammary gland development, resulting in significant alterations in the gland morphology, inducing intraductal hyperplasia that could be associated with an increased BCR [162,163]. The normal-like human breast epithelial cell line, MCF-10F, after exposure to BPA, showed an increased expression of breast cancer genes *BRCA1/2*, *BRCA1* associated RING domain 1 (*BARD1*), choline transporter-like protein (*CtlP*), RAD51 recombinase (*RAD51*), and BRCA1/2-containing complex subunit 3 (*BRCC3*), which are all involved in DNA repair, as well as the silencing of programmed cell death protein 5 (*PDCD5*) and Bcl-2-like 11 (*BCL2L11* (BIM)), which are involved in apoptosis [164].

For example, the breasts are particularly susceptible to polycyclic aromatic hydrocarbons (PAHs) that can affect cell morphology, cell division, growth, and repair, cell–cell junctions, and the number of p53 mutations [165]. Moreover, Korsh et al. (2015) investigated the link between PAHs and BC based on the use of biomarkers in measuring PAH-DNA adducts to assess the exposure level [165]. Polychlorinated biphenyls (PCBs) are persistent industrial pollutants that have been linked to BC progression [166]. Thus, many authors concluded that early life exposure to PCBs is a factor of BCR [12,167,168]. The highly reactive PCB metabolite, 2,3,5-trichloro-6-phenyl-[1,4]-benzoquinone (PCB29-pQ), induces metastasis of BC and increases cancer stem cell (CSC) biomarker expression, resulting in an increase in EMT in MDA-MB-231 BC cells; the Wnt/β-catenin pathway is also activated by PCB29-pQ, due to overproduction of ROS [166]. Many authors concluded that early life exposure to PCBs is a factor of BCR [12,167,168].

Phthalates, phenols, and parabens are considered non-persistent EDCs that have been associated with BC [169]. Biomarker concentrations of non-persistent EDCs tend to be higher among women than men, and among Black Americans compared to White Americans, especially based on inconsistent access to healthy food or use of certain products with higher concentration of phthalates, such hair relaxers and skin lightening topical products, that specifically target Black consumers [169]. Some phthalates that mimic estradiol may promote BC, as in the case of dibutyl phthalate (DBP) exposure, which is associated with a two-fold increase in the rate of ER+ BC [170].

Parabens, such as methylparaben (MeP), ethylparaben (EtP), propylparaben (PrP), and butylparaben (BuP), are a group of alkyl esters of the para-hydroxybenzoic acid esters [171] that can mimic estrogen in the body [172]. These chemicals are used as broad-spectrum antimicrobial preservatives in lotions/creams, skin foundation, eye makeup products, deodorants/perfumes, hair care products, shaving products, toothpastes, shampoos/conditioners, pharmaceuticals, textiles, clothes, and processed foods [173,174]. Parabens are absorbed by the dermal route or ingested and are systematically distributed and metabolized, being detected in human normal and tumoral tissues [175], hair [176], blood [171], saliva [177], breast milk [178,179], placenta [180], and urine [181]. Parabens can be found intact in the human breast [175] and preferentially accumulate in metastatic breast tumors compared to benign breast tumors [174]. Tapia et al. (2023) reported altered ER target gene expression and cell viability that was paraben- and cell line-specific [172].

**Table 1:** Pathological effects of the exposure to ECDs.

EDCs	Pathological Effects of EDC Exposure	References
DES	in utero exposure dysregulates gene expression and transcriptional reprogramming in adult fibroblasts, ECM composition and collagen deposition in adult mammary gland, molecular alteration develops over time and contributes to increased BCR in adulthood, induces epigenetic alterations/epimutations with intergenerational/transgenerational effects	[157,182]
PAHs (BaP and DB(ah)A)	in mammary gland, affect cellular morphology, cell-cell junctions, division, growth, repair, and number of p53 mutations, increase EVs production, changes in exosome content and gene expression control	[99,165]
BPA and other bisphenols (AF, F, S) and TBBPA	affect mammary gland development, resulting in precancerous and cancerous lesions in adulthood, exert estrogenic effects, activate the expression of genes associated with cell proliferation and BC; associated with EMT and BC progression; activate VEGF associated with angiogenesis, MAPK signaling pathway, Wnt/β-catenin pathway, STAT3 signaling, and DNA repair; induce changes in genes associated with apoptosis and DNA methylation; inactivate p53; increase expression of *BRCA1/2*, *BARD1*, *CtlP*, *RAD51*, and *BRCC3* involved in DNA repair; downregulate *PDCD5* and *BCL2L11* involved in apoptosis	[103,155,162,163,164,183,184]
Phtalates (DBP)	mimic estradiol, interact with ER and PR, promote BC, especially ER+ BC, interfere with DNA methylation and DNA damage	[170,185]
PCBs (PCB-153, PCB-180, PCB29-pQ)	BC cell proliferation by regulating ERK1/2 activation; induce cancer cell stemness and EMT via Wnt/β-catenin signaling	[166,184]
Organochlorine insecticides (DDT)	increase in utero BCR, BC progression by interfering with androgen signaling pathways, BC cells proliferation, negative effects on OS	[184,186]
Parabens (MeP, EtP, PrP, BuP) and their metabolites	promote protumorigenic effects in BC; modulate local estrogen-converting enzymes and increase local estrogen levels; cross-talk with HER2 pathway and affect ER signaling to increase pro-oncogenic c-Myc expression in ER+/HER2+ BC cells; alter ER target gene expression and cell viability	[172,173,181]

## 6. Conclusions

We are living in close interaction with a cocktail of man-made and natural xenobiotics. We are experiencing a wide spectrum of exposure during our lifetime, including the effects of xenobiotics on gametogenesis and gametes that undergo fertilization as the starting point of individual development and, moreover, in utero exposure that can initiate BC development. We are what we eat, we are what we breathe, and we are what we live. Most xenobiotics are metabolized or/and bioaccumulate and biomagnify in our tissues and cells, including breast tissues, so xenobiotic metabolism can play an important role in BC initiation and progression. This review pointed out the main mechanisms involved in the absorption, distribution, metabolism, bioaccumulation, biomagnification toxicity, and excretion of xenobiotics associated with BC risk, incidence, mortality, initiation, and progression. This association necessitates more valuable explanations at the biomolecular level to highlight the effects of genotoxic and epigenetic carcinogens. However, the accumulated xenobiotics, including their metabolites that arise as a consequence of biotransformation phases, such as heavy metals, endocrine-disrupting chemicals, or food contaminants, as well as a plethora of biomarkers of exposure, can be detected in breast tumoral tissues, adipose tissue, hair, blood, saliva, breast milk, placenta, and urine. In BC tissue biopsies and non-invasive liquid biopsies, xenobiotic exposure has been associated with changes in breast tissue composition and breast cell morphology, genomic instability, DNA damage, alteration of DNA repair, epimutations and epigenetic regulation, cell migration and invasion, angiogenesis, anti-apoptosis, cell adhesion, and cytoskeletal rearrangements, OS and ROS, metabolic reprogramming, immune regulation and metaflammation, membrane transport and signaling, extracellular matrix (ECM) and tumor microenvironment (TME) modifications, or extracellular vesicle (EV) production and content, with consequences in intercellular communication. At a biologic pathway level, most xenobiotics interact with endocrine signaling, adipogenesis, angiogenesis, DNA repair, inflammatory response, IGF-1 and NF-κB signaling, epithelial–mesenchymal transition (EMT), Wnt/β-catenin pathway, PI3K/Akt signaling, fatty acid metabolism (FAM) and glycolysis, MAPK, STAT3, p53 pathway, MYC targets, xenobiotic metabolism, and other cancer-related pathways. Fortunately, in our food, there are also many bioactive compounds with anti-tumor potential, which re-induce apoptosis by activation of caspases or target multiple signaling pathways, such as EMT migration-related pathway, Akt/NF-βB cell survival pathway, or p53 tumor suppressor signaling, that allow for cell survival, proliferation, growth, and metastatic progression of BC cells.

Consequently, BC can be characterized as an environmental disease or an ecological disorder. Evidence for BC risk suggests that food-borne chemical carcinogens, air pollution, ionizing radiation, and socioeconomic status are closely related to breast carcinogenesis. Thus, exposomics and the exposome concept are based on the diversity and range of exposures to physical factors, synthetic chemicals, dietary components, and psychosocial stressors, as well as their associated biological responses. Advances in molecular sciences and analytical techniques based on high-throughput sequencing and mass spectrometry (MS) have generated multi-omics data that can be successfully used to understand the complexity of molecular mechanisms involved in BC exposomics. Thus, environmental toxicogenomics, epigenomics, and interactomics, as well as nutrigenomics and nutriproteomics, metagenomics, micromiRomics, and nutrimiRomics are several new omics fields related to BC exposomics, which can contribute to molecular characterization of the complex relationship between the human body, environmental exposure, and breast cancer.

## Data Availability

Data sharing is not applicable.

## Abbreviations

BaP—benz(a)pyrene; BCR—breast cancer risk; BPA—bisphenol A; BuP—butylparaben; DB(ah)A—dibenz(ah)anthracene; DBP—dibutyl phthalate; DES—diethylstilbestrol; DDT—dichlorodiphenyltrichloroethane; ECM—extracellular matrix; EDC—endocrine-disrupting chemicals; EMT—epithelial–mesenchymal transition; ER—estrogen receptor; EtP—ethylparaben; EVs—extracellular vesicles; MeP—methylparaben; OS—overall survival; PAHs—polycyclic aromatic hydrocarbons; PCBs—polychlorinated biphenyls; PCB-29-pQ—polychlorinated biphenyl quinone; PR—progesterone receptor; PrP—propylparaben; TBBPA—tetrabromobisphenol A; VEGF—vascular endothelial growth factor.

## References

[1] Lynn H., Ward D., Burton D., Day J., Craig A., Parnell M., Dimmer C. (2005). Breast Cancer: An Environmental Disease. The Case for Primary Prevention. https://www.researchgate.net/publication/275209371_Breast_Cancer_an_environmental_disease_The_case_for_primary_prevention.

[2] Hiatt R.A., Brody J.G. (2018). Environmental Determinants of Breast Cancer. Annu. Rev. Public Health.

[3] Neagu A.-N., Whitham D., Bruno P., Arshad A., Seymour L., Morrissiey H., Hukovic A.I., Darie C.C. (2024). Onco-Breastomics: An Eco-Evo-Devo Holistic Approach. Int. J. Mol. Sci..

[4] Mathipa E.R., Semuli Q.K. (2015). We are what we eat. Rethinking Teaching and Learning in the 21st Century, Proceedings of the South Africa International Conference on Education, Pretoria, South Africa, 21–23 September 2015.

[5] Pretty J. (2004). We are what we eat. New Sci..

[6] Rumiati R.I., Foroni F. (2016). We are what we eat: How food is represented in our mind/brain. Psychon. Bull. Rev..

[7] Hull S.C., Charles J., Caplan A.L. (2023). Are We What We Eat? The Moral Imperative of the Medical Profession to Promote Plant-Based Nutrition. Am. J. Cardiol..

[8] Rajkhowa S. (2021). “ARE WE WHAT WE EAT?”: Understanding Identities through Food. Master’s Thesis.

[9] Miller M.R., Shah A.S.V., Newby D.E. (2020). We all breathe the same air … and we are all mortal. Cardiovasc. Res..

[10] Sorin Mihalache A. (2018). How do We Live and what is the World We Live in Like? Some Possible Neuroscientific Evaluations on the Anthropology of the Spiritual Life in the Context of the Contemporary Society. Glob. J. Anthropol. Res..

[11] Strumylaitė L., Mechonošina K., Tamašauskas Š. (2010). Environmental factors and breast cancer. Medicina.

[12] Leng L., Li J., Luo X.-M., Kim J.-Y., Li Y.-M., Guo X.-M., Chen X., Yang Q.-Y., Li G., Tang N.-J. (2016). Polychlorinated biphenyls and breast cancer: A congener-specific meta-analysis. Environ. Int..

[13] Bucher M.L., Anderson F.L., Lai Y., Dicent J., Miller G.W., Zota A.R. (2023). Exposomics as a tool to investigate differences in health and disease by sex and gender. Exposome.

[14] Koual M., Tomkiewicz C., Cano-Sancho G., Antignac J.-P., Bats A.-S., Coumoul X. (2020). Environmental chemicals, breast cancer progression and drug resistance. Environ. Health.

[15] Feng Y., Spezia M., Huang S., Yuan C., Zeng Z., Zhang L., Ji X., Liu W., Huang B., Luo W. (2018). Breast cancer development and progression: Risk factors, cancer stem cells, signaling pathways, genomics, and molecular pathogenesis. Genes Dis..

[16] Terry M.B., Michels K.B., Brody J.G., Byrne C., Chen S., Jerry D.J., Malecki K.M.C., Martin M.B., Miller R.L., on behalf of Breast Cancer and the Environment Research Program (BCERP) (2019). Environmental exposures during windows of susceptibility for breast cancer: A framework for prevention research. Breast Cancer Res..

[17] Hiatt R.A., Beyeler N. (2023). Women’s cancers and climate change. Int. J. Gynecol. Obstet..

[18] Shih Y.-W., Hung C.-S., Huang C.-C., Chou K.-R., Niu S.-F., Chan S., Tsai H.-T. (2020). The Association Between Smartphone Use and Breast Cancer Risk Among Taiwanese Women: A Case-Control Study. Cancer Manag. Res..

[19] Garcia-Saenz A., De Miguel A.S., Espinosa A. (2018). Evaluating the Association between Artificial Light-at-Night Exposure and Breast and Prostate Cancer Risk in Spain (MCC-Spain Study). Environ. Health Perspect..

[20] Hinchliffe A., Kogevinas M., Pérez-Gómez B., Ardanaz E., Amiano P., Marcos-Delgado A., Castaño-Vinyals G., Llorca J., Moreno V., Alguacil J. (2021). Occupational Heat Exposure and Breast Cancer Risk in the MCC-Spain Study. Cancer Epidemiol. Biomark. Prev..

[21] Gera R., Mokbel R., Igor I., Mokbel K. (2018). Does the Use of Hair Dyes Increase the Risk of Developing Breast Cancer? A Meta-analysis and Review of the Literature. Anticancer Res..

[22] Jones M.E., Schoemaker M.J., Wright L.B., Ashworth A., Swerdlow A.J. (2017). Smoking and risk of breast cancer in the Generations Study cohort. Breast Cancer Res..

[23] Huynh D., Huang J., Le L.T.T., Liu D., Liu C., Pham K., Wang H. (2020). Electronic cigarettes promotes the lung colonization of human breast cancer in NOD-SCID-Gamma mice. Int. J. Clin. Exp. Pathol..

[24] Shih Y.W., O’brien A.P., Hung C.S., Chen K.H., Hou W.H., Tsai H.T. (2021). Exposure to radiofrequency radiation increases the risk of breast cancer: A systematic review and meta-analysis. Exp. Ther. Med..

[25] Mortazavi A.R., Mortazavi S.M.J. (2018). Women with hereditary breast cancer predispositions should avoid using their smartphones, tablets and laptops at night. Iran. J. Basic Med. Sci..

[26] Vinogradova Y., Coupland C., Hippisley-Cox J. (2020). Use of hormone replacement therapy and risk of breast cancer: Nested case-control studies using the QResearch and CPRD databases. BMJ.

[27] de Blok C.J.M., Wiepjes C.M., Nota N.M., van Engelen K., Adank M.A., Dreijerink K.M.A., Barbé E., Konings I.R.H.M., den Heijer M. (2019). Breast cancer risk in transgender people receiving hormone treatment: Nationwide cohort study in The Netherlands. BMJ.

[28] Sørensen M., Poulsen A.H., Kroman N., Hvidtfeldt U.A., Thacher J.D., Roswall N., Brandt J., Frohn L.M., Jensen S.S., Levin G. (2021). Road and railway noise and risk for breast cancer: A nationwide study covering Denmark. Environ. Res..

[29] Andersen Z.J., Jørgensen J.T., Elsborg L., Lophaven S.N., Backalarz C., Laursen J.E., Pedersen T.H., Simonsen M.K., Bräuner E.V., Lynge E. (2018). Long-term exposure to road traffic noise and incidence of breast cancer: A cohort study. Breast Cancer Res..

[30] Xiang P., Wang K., Bi J., Li M., He R.-W., Cui D., Ma L.Q. (2020). Organic extract of indoor dust induces estrogen-like effects in human breast cancer cells. Sci. Total Environ..

[31] Afzal S., Fiaz K., Noor A., Sindhu A.S., Hanif A., Bibi A., Asad M., Nawaz S., Zafar S., Ayub S. (2022). Interrelated Oncogenic Viruses and Breast Cancer. Front. Mol. Biosci..

[32] Vermeulen R., Schymanski E.L., Barabási A.-L., Miller G.W. (2020). The exposome and health: Where chemistry meets biology. Science.

[33] Ziegler R.G., Hoover R.N., Pike M.C., Hildesheim A., Nomura A.M.Y., West D.W., Wu-Williams A.H., Kolonel L.N., Horn-Ross P.L., Rosenthal J.F. (1993). Migration Patterns and Breast Cancer Risk in Asian-American Women. J. Natl. Cancer Inst..

[34] Christ A., Latz E. (2019). The Western lifestyle has lasting effects on metaflammation. Nat. Rev. Immunol..

[35] Danforth D.N. (2021). The Role of Chronic Inflammation in the Development of Breast Cancer. Cancers.

[36] Itoh H., Ueda M., Suzuki M., Kohmura-Kobayashi Y. (2022). Developmental Origins of Metaflammation; A Bridge to the Future Between the DOHaD Theory and Evolutionary Biology. Front. Endocrinol..

[37] Wani B., Aziz S.A., Ganaie M.A., Mir M.H. (2017). Metabolic Syndrome and Breast Cancer Risk. Indian J. Med. Paediatr. Oncol..

[38] Dong S., Wang Z., Shen K., Chen X. (2021). Metabolic Syndrome and Breast Cancer: Prevalence, Treatment Response, and Prognosis. Front. Oncol..

[39] Choi I.Y., Chun S., Shin D.W., Han K., Jeon K.H., Yu J., Chae B.J., Suh M., Park Y.-M. (2021). Changes in Metabolic Syndrome Status and Breast Cancer Risk: A Nationwide Cohort Study. Cancers.

[40] Starek-Świechowicz B., Budziszewska B., Starek A. (2021). Endogenous estrogens—Breast cancer and chemoprevention. Pharmacol. Rep..

[41] Autrup H., Barile F.A., Berry S.C., Blaauboer B.J., Boobis A., Bolt H., Borgert C.J., Dekant W., Dietrich D., Domingo J.L. (2020). Human exposure to synthetic endocrine disrupting chemicals (S-EDCs) is generally negligible as compared to natural compounds with higher or comparable endocrine activity: How to evaluate the risk of the S-EDCs?. Arch. Toxicol..

[42] Kazemi A., Barati-Boldaji R., Soltani S., Mohammadipoor N., Esmaeilinezhad Z., Clark C.C.T., Babajafari S., Akbarzadeh M. (2021). Intake of Various Food Groups and Risk of Breast Cancer: A Systematic Review and Dose-Response Meta-Analysis of Prospective Studies. Adv. Nutr..

[43] Merugu N.K., Manapuram S., Chakraborty T., Karanam S.K., Imandi S.B. (2023). Mutagens in commercial food processing and its microbial transformation. Food Sci. Biotechnol..

[44] Thakkar N., Bin Shin Y., Sung H.-K. (2022). Nutritional Regulation of Mammary Tumor Microenvironment. Front. Cell Dev. Biol..

[45] Wiseman M. (2008). The Second World Cancer Research Fund/American Institute for Cancer Research Expert Report. Food, Nutrition, Physical Activity, and the Prevention of Cancer: A Global Perspective: Nutrition Society and BAPEN Medical Symposium on ‘Nutrition support in cancer therapy’. Proc. Nutr. Soc..

[46] Wan M.L.Y., Co V.A., El-Nezami H. (2022). Endocrine disrupting chemicals and breast cancer: A systematic review of epidemiological studies. Crit. Rev. Food Sci. Nutr..

[47] Darbre P.D., Vandenberg L.N., Turgeon J.L. (2021). Chapter Thirteen—Endocrine disrupting chemicals and breast cancer cells. Advances in Pharmacology.

[48] Jacobs I., Taljaard-Krugell C., Wicks M., Cubasch H., Joffe M., Laubscher R., Romieu I., Biessy C., Rinaldi S., Huybrechts I. (2021). Dietary Patterns and Breast Cancer Risk in Black Urban South African Women: The SABC Study. Nutrients.

[49] McDonald J.A., Goyal A., Terry M.B. (2013). Alcohol Intake and Breast Cancer Risk: Weighing the Overall Evidence. Curr. Breast Cancer Rep..

[50] Wang Y., Xu M., Ke Z.-J., Luo J. (2017). Cellular and Molecular Mechanism Underlying Alcohol-induced Aggressiveness of Breast Cancer. Pharmacol. Res..

[51] VoPham T., Bertrand K.A., Jones R.R., Deziel N.C., DuPré N.C., James P., Liu Y., Vieira V.M., Tamimi R.M., Hart J.E. (2020). Dioxin exposure and breast cancer risk in a prospective cohort study. Environ. Res..

[52] Lee P.M.Y., Chan W.C., Kwok C.C.-H., Wu C., Law S.-H., Tsang K.-H., Yu W.-C., Yeung Y.-C., Chang L.D.J., Wong C.K.M. (2019). Associations between Coffee Products and Breast Cancer Risk: A Case-Control study in Hong Kong Chinese Women. Sci. Rep..

[53] Fiolet T., Srour B., Sellem L., Kesse-Guyot E., Allès B., Méjean C., Deschasaux M., Fassier P., Latino-Martel P., Touvier M. (2018). Consumption of ultra-processed foods and cancer risk: Results from NutriNet-Santé prospective cohort. BMJ.

[54] Lo J.J., Park Y.-M., Sinha R., Sandler D.P. (2019). Association between meat consumption and risk of breast cancer: Findings from the Sister Study. Int. J. Cancer.

[55] Chazelas E., Srour B., Desmetz E., Kesse-Guyot E., Julia C., Deschamps V., Druesne-Pecollo N., Galan P., Hercberg S., Latino-Martel P. (2019). Sugary drink consumption and risk of cancer: Results from NutriNet-Santé prospective cohort. BMJ.

[56] Eve L., Fervers B., Le Romancer M., Etienne-Selloum N. (2020). Exposure to Endocrine Disrupting Chemicals and Risk of Breast Cancer. Int. J. Mol. Sci..

[57] Shen J., Liao Y., Hopper J.L., Goldberg M., Santella R.M., Terry M.B. (2017). Dependence of cancer risk from environmental exposures on underlying genetic susceptibility: An illustration with polycyclic aromatic hydrocarbons and breast cancer. Br. J. Cancer.

[58] Keren Y., Magnezi R., Carmon M., Amitai Y. (2020). Investigation of the Association between Drinking Water Habits and the Occurrence of Women Breast Cancer. Int. J. Environ. Res. Public Health.

[59] Maltarollo V.G., Gertrudes J.C., Oliveira P.R., Honorio K.M. (2015). Applying machine learning techniques for ADME-Tox prediction: A review. Expert Opin. Drug Metab. Toxicol..

[60] Neagu A.-N., Whitham D., Bruno P., Morrissiey H., Darie C.A., Darie C.C. (2023). Omics-Based Investigations of Breast Cancer. Molecules.

[61] Rasool R., Ullah I., Mubeen B., Alshehri S., Imam S.S., Ghoneim M.M., Alzarea S.I., Al-Abbasi F.A., Murtaza B.N., Kazmi I. (2022). Theranostic Interpolation of Genomic Instability in Breast Cancer. Int. J. Mol. Sci..

[62] Portugal J., Mansilla S., Piña B. (2022). Perspectives on the Use of Toxicogenomics to Assess Environmental Risk. Front. Biosci..

[63] Santibáñez-Andrade M., Quezada-Maldonado E.M., Osornio-Vargas Á., Sánchez-Pérez Y., García-Cuellar C.M. (2017). Air pollution and genomic instability: The role of particulate matter in lung carcinogenesis. Environ. Pollut..

[64] Coppedè F. (2023). Genes and the Environment in Cancer: Focus on Environmentally Induced DNA Methylation Changes. Cancers.

[65] Offiong N.-A.O., Edet J.B., Shaibu S.E., Akan N.E., Atakpa E.O., Sanganyado E., Okop I.J., Benson N.U., Okoh A. (2023). Metagenomics: An emerging tool for the chemistry of environmental remediation. Front. Environ. Chem..

[66] Zhang Y., Keerthisinghe T.P., Han Y., Liu M., Wanjaya E.R., Fang M. (2018). “Cocktail” of Xenobiotics at Human Relevant Levels Reshapes the Gut Bacterial Metabolome in a Species-Specific Manner. Environ. Sci. Technol..

[67] Kartti S., Bendani H., Boumajdi N., Bouricha E.M., Zarrik O., EL Agouri H., Fokar M., Aghlallou Y., EL Jaoudi R., Belyamani L. (2023). Metagenomics Analysis of Breast Microbiome Highlights the Abundance of Rothia Genus in Tumor Tissues. J. Pers. Med..

[68] Iida M., Takemoto K. (2018). A network biology-based approach to evaluating the effect of environmental contaminants on human interactome and diseases. Ecotoxicol. Environ. Saf..

[69] Moslehi R., Stagnar C., Srinivasan S., Radziszowski P., Carpenter D.O. (2021). The possible role of arsenic and gene-arsenic interactions in susceptibility to breast cancer: A systematic review. Rev. Environ. Health.

[70] Lim CJ C., Lim PP C., Pizarro RR M., Segocio HG B., Ratta K., Dable-Tupas G., Egbuna C. (2023). 8—Nutrigenomics in the management and prevention of cancer. Role of Nutrigenomics in Modern-Day Healthcare and Drug Discovery.

[71] Sellami M., Bragazzi N.L. (2020). Nutrigenomics and Breast Cancer: State-of-Art, Future Perspectives and Insights for Prevention. Nutrients.

[72] Schraml E., Hackl M., Grillari J. (2017). MicroRNAs and toxicology: A love marriage. Toxicol. Rep..

[73] Singh R., Mo Y.-Y. (2013). Role of microRNAs in breast cancer. Cancer Biol. Ther..

[74] Balasubramanian S., Gunasekaran K., Sasidharan S., Mathan V.J., Perumal E. (2020). MicroRNAs and Xenobiotic Toxicity: An Overview. Toxicol. Rep..

[75] Dai T., Zhao X., Li Y., Yu L., Li Y., Zhou X., Gong Q. (2020). miR-423 Promotes Breast Cancer Invasion by Activating NF-κB Signaling. OncoTargets Ther..

[76] Morales-Pison S., Jara L., Carrasco V., Gutiérrez-Vera C., Reyes J.M., Gonzalez-Hormazabal P., Carreño L.J., Tapia J.C., Contreras H.R. (2022). Genetic Variation in MicroRNA-423 Promotes Proliferation, Migration, Invasion, and Chemoresistance in Breast Cancer Cells. Int. J. Mol. Sci..

[77] Farahani M., Rezaei-Tavirani M., Arjmand B. (2021). A systematic review of microRNA expression studies with exposure to bisphenol A. J. Appl. Toxicol..

[78] Quintanilha B.J., Reis B.Z., Duarte G.B.S., Cozzolino S.M.F., Rogero M.M. (2017). Nutrimiromics: Role of microRNAs and Nutrition in Modulating Inflammation and Chronic Diseases. Nutrients.

[79] Venkatadri R., Muni T., Iyer A.K.V., Yakisich J.S., Azad N. (2016). Role of apoptosis-related miRNAs in resveratrol-induced breast cancer cell death. Cell Death Dis..

[80] Santoro K.L., Yakah W., Singh P., Ramiro-Cortijo D., Medina-Morales E., Freedman S.D., Martin C.R. (2022). Acetaminophen and Xenobiotic Metabolites in Human Milk and the Development of Bronchopulmonary Dysplasia and Retinopathy of Prematurity in a Cohort of Extremely Preterm Infants. J. Pediatr..

[81] Rosen M., Bulucea C., Brindusa C., Mastorakis N. (2012). Approaching Resonant Absorption of Environmental Xenobiotics Harmonic Oscillation by Linear Structures. Sustainability.

[82] Idle J.R., Gonzalez F.J. (2007). Metabolomics. Cell Metab..

[83] Williams J.A., Phillips D.H. (2000). Mammary Expression of Xenobiotic Metabolizing Enzymes and Their Potential Role in Breast Cancer. Cancer Res..

[84] Swift L.H., Golsteyn R.M. (2014). Genotoxic Anti-Cancer Agents and Their Relationship to DNA Damage, Mitosis, and Checkpoint Adaptation in Proliferating Cancer Cells. Int. J. Mol. Sci..

[85] Zahreddine H., Borden K.L.B. (2013). Mechanisms and insights into drug resistance in cancer. Front. Pharmacol..

[86] Rylander C., Veierød M.B., Weiderpass E., Lund E., Sandanger T.M. (2019). Use of skincare products and risk of cancer of the breast and endometrium: A prospective cohort study. Environ. Health.

[87] Filipiuc S.-I., Neagu A.-N., Uritu C.M., Tamba B.-I., Filipiuc L.-E., Tudorancea I.M., Boca A.N., Hâncu M.F., Porumb V., Bild W. (2023). The Skin and Natural Cannabinoids–Topical and Transdermal Applications. Pharmaceuticals.

[88] Andersen E.R., Eilertsen G., Myklebust A.M., Eriksen S. (2018). Women’s experience of acute skin toxicity following radiation therapy in breast cancer. J. Multidiscip. Healthc..

[89] Gregoire A.M., VoPham T., Laden F., Yarosh R., O’Brien K.M., Sandler D.P., White A.J. (2022). Residential ultraviolet radiation and breast cancer risk in a large prospective cohort. Environ. Int..

[90] Chee Y.C., Pahnke J., Bunte R., Adsool V.A., Madan B., Virshup D.M. (2018). Intrinsic Xenobiotic Resistance of the Intestinal Stem Cell Niche. Dev. Cell.

[91] Wen L., Han Z. (2021). Identification and validation of xenobiotic metabolism-associated prognostic signature based on five genes to evaluate immune microenvironment in colon cancer. J. Gastrointest. Oncol..

[92] White A.J., Gregoire A.M., Fisher J.A., Medgyesi D.N., Li L., Koutrakis P., Sandler D.P., Jones R.R. (2022). Exposure to Particle Radioactivity and Breast Cancer Risk in the Sister Study: A U.S.-Wide Prospective Cohort. Environ. Health Perspect..

[93] Olesiejuk K., Chałubiński M. (2023). How does particulate air pollution affect barrier functions and inflammatory activity of lung vascular endothelium?. Allergy.

[94] Smotherman C., Sprague B., Datta S., Braithwaite D., Qin H., Yaghjyan L. (2023). Association of air pollution with postmenopausal breast cancer risk in UK Biobank. Breast Cancer Res..

[95] Moriceau M.-A., Cano-Sancho G., Kim M., Coumoul X., Emond C., Arrebola J.-P., Antignac J.-P., Audouze K., Rousselle C. (2023). Partitioning of Persistent Organic Pollutants between Adipose Tissue and Serum in Human Studies. Toxics.

[96] Ish J.L., Abubakar M., Fan S., Jones R.R., Niehoff N.M., Henry J.E., Gierach G.L., White A.J. (2023). Outdoor air pollution and histologic composition of normal breast tissue. Environ. Int..

[97] Segovia-Mendoza M., de León C.T.G., García-Becerra R., Ambrosio J., Nava-Castro K.E., Morales-Montor J. (2020). The chemical environmental pollutants BPA and BPS induce alterations of the proteomic profile of different phenotypes of human breast cancer cells: A proposed interactome. Environ. Res..

[98] Yuan J., Liu Y., Wang J., Zhao Y., Li K., Jing Y., Zhang X., Liu Q., Geng X., Li G. (2018). Long-term Persistent Organic Pollutants Exposure Induced Telomere Dysfunction and Senescence-Associated Secretary Phenotype. J. Gerontol. Ser. A.

[99] Montjean D., Neyroud A.-S., Yefimova M.G., Benkhalifa M., Cabry R., Ravel C. (2022). Impact of Endocrine Disruptors upon Non-Genetic Inheritance. Int. J. Mol. Sci..

[100] Dungen M.W.v.D., Murk A.J., Kok D.E., Steegenga W.T. (2017). Persistent organic pollutants alter DNA methylation during human adipocyte differentiation. Toxicol. Vitr..

[101] Wielsøe M., Tarantini L., Bollati V., Long M., Bonefeld-Jørgensen E.C. (2020). DNA methylation level in blood and relations to breast cancer, risk factors and environmental exposure in Greenlandic Inuit women. Basic Clin. Pharmacol. Toxicol..

[102] Zucchini-Pascal N., Peyre L., de Sousa G., Rahmani R. (2012). Organochlorine pesticides induce epithelial to mesenchymal transition of human primary cultured hepatocytes. Food Chem. Toxicol..

[103] Lee H.-M., Hwang K.-A., Choi K.-C. (2016). Diverse pathways of epithelial mesenchymal transition related with cancer progression and metastasis and potential effects of endocrine disrupting chemicals on epithelial mesenchymal transition process. Mol. Cell. Endocrinol..

[104] Zhang X.-L., Wang H.-S., Liu N., Ge L.-C. (2015). Bisphenol A stimulates the epithelial mesenchymal transition of estrogen negative breast cancer cells via FOXA1 signals. Arch. Biochem. Biophys..

[105] Johnson C.H., Patterson A.D., Idle J.R., Gonzalez F.J. (2012). Xenobiotic Metabolomics: Major Impact on the Metabolome. Annu. Rev. Pharmacol. Toxicol..

[106] Bièche I., Girault I., Urbain E., Tozlu S., Lidereau R. (2004). Relationship between intratumoral expression of genes coding for xenobiotic-metabolizing enzymes and benefit from adjuvant tamoxifen in estrogen receptor alpha-positive postmenopausal breast carcinoma. Breast Cancer Res..

[107] Li Y., Steppi A., Zhou Y., Mao F., Miller P.C., He M.M., Zhao T., Sun Q., Zhang J. (2017). Tumoral expression of drug and xenobiotic metabolizing enzymes in breast cancer patients of different ethnicities with implications to personalized medicine. Sci. Rep..

[108] Men Y., Li L., Zhang F., Kong X., Zhang W., Hao C., Wang G. (2020). Evaluation of heavy metals and metabolites in the urine of patients with breast cancer. Oncol. Lett..

[109] Tarhonska K., Janasik B., Roszak J., Kowalczyk K., Lesicka M., Reszka E., Wieczorek E., Braun M., Kolacinska-Wow A., Skokowski J. (2023). Environmental exposure to cadmium in breast cancer—Association with the Warburg effect and sensitivity to tamoxifen. Biomed. Pharmacother..

[110] Gadzała-Kopciuch R., Pajewska-Szmyt M., Buszewski B., Baranowska I. (2022). Human Milk and Xenobiotics. Handbook of Bioanalytics.

[111] Mead M. (2008). Contaminants in Human Milk: Weighing the Risks against the Benefits of Breastfeeding. Environ. Health Perspect..

[112] Ennour-Idrissi K., Ayotte P., Diorio C. (2019). Persistent Organic Pollutants and Breast Cancer: A Systematic Review and Critical Appraisal of the Literature. Cancers.

[113] Jackson E., Shoemaker R., Larian N., Cassis L. (2017). Adipose Tissue as a Site of Toxin Accumulation. Compr. Physiol..

[114] Lee Y., Kim K., Jacobs D.R., Lee D. (2017). Persistent organic pollutants in adipose tissue should be considered in obesity research. Obes. Rev..

[115] Zhang A., Wang R., Liu Q., Yang Z., Lin X., Pang J., Li X., Wang D., He J., Li J. (2023). Breast adipose metabolites mediates the association of tetrabromobisphenol a with breast cancer: A case-control study in Chinese population. Environ. Pollut..

[116] Liu T., Liang X., Lei C., Huang Q., Song W., Fang R., Li C., Li X., Mo H., Sun N. (2020). High-Fat Diet Affects Heavy Metal Accumulation and Toxicity to Mice Liver and Kidney Probably via Gut Microbiota. Front. Microbiol..

[117] Romaniuk A., Lyndin M., Sikora V., Lyndina Y., Romaniuk S., Sikora K. (2017). Heavy metals effect on breast cancer progression. J. Occup. Med. Toxicol..

[118] Wang X., Mukherjee B., Park S.K. (2018). Associations of cumulative exposure to heavy metal mixtures with obesity and its comorbidities among U.S. adults in NHANES 2003–2014. Environ. Int..

[119] Teo P.S., van Dam R.M., Whitton C., Tan L.W.L., Forde C.G. (2021). Consumption of Foods With Higher Energy Intake Rates is Associated With Greater Energy Intake, Adiposity, and Cardiovascular Risk Factors in Adults. J. Nutr..

[120] Bernard J.J., Wellberg E.A. (2021). The Tumor Promotional Role of Adipocytes in the Breast Cancer Microenvironment and Macroenvironment. Am. J. Pathol..

[121] Kothari C., Diorio C., Durocher F. (2020). The Importance of Breast Adipose Tissue in Breast Cancer. Int. J. Mol. Sci..

[122] Tiwari S., Sapkota N., Han Z. (2022). Effect of fasting on cancer: A narrative review of scientific evidence. Cancer Sci..

[123] Anemoulis M., Vlastos A., Kachtsidis V., Karras S.N. (2023). Intermittent Fasting in Breast Cancer: A Systematic Review and Critical Update of Available Studies. Nutrients.

[124] Melnik B.C., John S.M., Schmitz G. (2011). Over-stimulation of insulin/IGF-1 signaling by Western diet may promote diseases of civilization: Lessons learnt from Laron syndrome. Nutr. Metab..

[125] Christopoulos P.F., Msaouel P., Koutsilieris M. (2015). The role of the insulin-like growth factor-1 system in breast cancer. Mol. Cancer.

[126] Cunha S.C., Menezes-Sousa D., Mello F.V., Miranda J.A., Fogaca F.H., Alonso M.B., Torres J.P.M., Fernandes J.O. (2022). Survey on endocrine-disrupting chemicals in seafood: Occurrence and distribution. Environ. Res..

[127] Law A.Y.S., Wei X., Zhang X., Mak N.K., Cheung K.C., Wong M.H., Giesy J.P., Wong C.K.C. (2012). Biological analysis of endocrine-disrupting chemicals in animal meats from the Pearl River Delta, China. J. Expo. Sci. Environ. Epidemiol..

[128] Mukherjee R., Pandya P., Baxi D., Ramachandran A.V. (2021). Endocrine Disruptors–‘Food’ for Thought. Proc. Zool. Soc..

[129] Chang J., Zhou J., Gao M., Zhang H., Wang T. (2022). Research Advances in the Analysis of Estrogenic Endocrine Disrupting Compounds in Milk and Dairy Products. Foods.

[130] Djedjibegovic J., Marjanovic A., Tahirovic D., Caklovica K., Turalic A., Lugusic A., Omeragic E., Sober M., Caklovica F. (2020). Heavy metals in commercial fish and seafood products and risk assessment in adult population in Bosnia and Herzegovina. Sci. Rep..

[131] Nindrea R.D., Aryandono T., Lazuardi L., Dwiprahasto I. (2019). Protective Effect of Omega-3 Fatty Acids in Fish Consumption Against Breast Cancer in Asian Patients: A Meta-Analysis. Asian Pac. J. Cancer Prev..

[132] Rodgers K.M., Udesky J.O., Rudel R.A., Brody J.G. (2018). Environmental chemicals and breast cancer: An updated review of epidemiological literature informed by biological mechanisms. Environ. Res..

[133] Meng Q., Gao B., Goldberg I.D., Rosen E.M., Fan S. (2000). Stimulation of Cell Invasion and Migration by Alcohol in Breast Cancer Cells. Biochem. Biophys. Res. Commun..

[134] Muniraj N., Siddharth S., Sharma D. (2019). Bioactive Compounds: Multi-Targeting Silver Bullets for Preventing and Treating Breast Cancer. Cancers.

[135] Carlos-Reyes Á., López-González J.S., Meneses-Flores M., Gallardo-Rincón D., Ruíz-García E., Marchat L.A., la Vega H.A.-D., de la Cruz O.N.H., López-Camarillo C. (2019). Dietary Compounds as Epigenetic Modulating Agents in Cancer. Front. Genet..

[136] Björner S., Rosendahl A.H., Tryggvadottir H., Simonsson M., Jirström K., Borgquist S., Rose C., Ingvar C., Jernström H. (2018). Coffee Is Associated With Lower Breast Tumor Insulin-Like Growth Factor Receptor 1 Levels in Normal-Weight Patients and Improved Prognosis Following Tamoxifen or Radiotherapy Treatment. Front. Endocrinol..

[137] Hayakawa S., Ohishi T., Miyoshi N., Oishi Y., Nakamura Y., Isemura M. (2020). Anti-Cancer Effects of Green Tea Epigallocatchin-3-Gallate and Coffee Chlorogenic Acid. Molecules.

[138] Zeng A., Liang X., Zhu S., Liu C., Wang S., Zhang Q., Zhao J., Song L. (2021). Chlorogenic acid induces apoptosis, inhibits metastasis and improves antitumor immunity in breast cancer via the NF-κB signaling pathway. Oncol. Rep..

[139] Deus C.M., Serafim T.L., Magalhães-Novais S., Vilaça A., Moreira A.C., Sardão V.A., Cardoso S.M., Oliveira P.J. (2017). Sirtuin 1-dependent resveratrol cytotoxicity and pro-differentiation activity on breast cancer cells. Arch. Toxicol..

[140] Greenshields A.L., Doucette C.D., Sutton K.M., Madera L., Annan H., Yaffe P.B., Knickle A.F., Dong Z., Hoskin D.W. (2015). Piperine inhibits the growth and motility of triple-negative breast cancer cells. Cancer Lett..

[141] Peng C., Zeleznik O.A., Shutta K.H., Rosner B.A., Kraft P., Clish C.B., Stampfer M.J., Willett W.C., Tamimi R.M., Eliassen A.H. (2022). A Metabolomics Analysis of Circulating Carotenoids and Breast Cancer Risk. Cancer Epidemiol. Biomark. Prev..

[142] Maitisha G., Aimaiti M., An Z., Li X. (2021). Allicin induces cell cycle arrest and apoptosis of breast cancer cells in vitro via modulating the p53 pathway. Mol. Biol. Rep..

[143] Rosas-González V.C., Téllez-Bañuelos M.C., Hernández-Flores G., Bravo-Cuellar A., Aguilar-Lemarroy A., Jave-Suárez L.F., Haramati J., Solorzano-Ibarra F. (2020). Differential effects of alliin and allicin on apoptosis and senescence in luminal A and triple-negative breast cancer: Caspase, ΔΨm, and pro-apoptotic gene involvement. Fundam. Clin. Pharmacol..

[144] Schley P.D., Jijon H.B., Robinson L.E., Field C.J. (2005). Mechanisms of omega-3 fatty acid-induced growth inhibition in MDA-MB-231 human breast cancer cells. Breast Cancer Res. Treat..

[145] Annamalai J., Namasivayam V. (2015). Endocrine disrupting chemicals in the atmosphere: Their effects on humans and wildlife. Environ. Int..

[146] Gonsioroski A., Mourikes V.E., Flaws J.A. (2020). Endocrine Disruptors in Water and Their Effects on the Reproductive System. Int. J. Mol. Sci..

[147] Mnif W., Hassine AI H., Bouaziz A., Bartegi A., Thomas O., Roig B. (2011). Effect of Endocrine Disruptor Pesticides: A Review. Int. J. Environ. Res. Public Health.

[148] Iavicoli I., Fontana L., Bergamaschi A. (2009). The Effects of Metals as Endocrine Disruptors. J. Toxicol. Environ. Health Part B.

[149] Peivasteh-Roudsari L., Barzegar-Bafrouei R., Sharifi K.A., Azimisalim S., Karami M., Abedinzadeh S., Asadinezhad S., Tajdar-Oranj B., Mahdavi V., Alizadeh A.M. (2023). Origin, dietary exposure, and toxicity of endocrine-disrupting food chemical contaminants: A comprehensive review. Heliyon.

[150] Peinado F.M., Iribarne-Durán L.M., Ocón-Hernández O., Olea N., Artacho-Cordón F., Courtney M. (2020). Endocrine Disrupting Chemicals in Cosmetics and Personal Care Products and Risk of Endometriosis. Endometriosis.

[151] Monneret C. (2017). What is an endocrine disruptor?. Comptes Rendus Biol..

[152] Marinello W.P., Patisaul H.B., Vandenberg L.N., Turgeon J.L. (2021). Chapter Nine—Endocrine disrupting chemicals (EDCs) and placental function: Impact on fetal brain development. Advances in Pharmacology.

[153] Schjenken J.E., Green E.S., Overduin T.S., Mah C.Y., Russell D.L., Robertson S.A. (2021). Endocrine Disruptor Compounds—A Cause of Impaired Immune Tolerance Driving Inflammatory Disorders of Pregnancy?. Front. Endocrinol..

[154] Alavian-Ghavanini A., Rüegg J. (2018). Understanding Epigenetic Effects of Endocrine Disrupting Chemicals: From Mechanisms to Novel Test Methods. Basic Clin. Pharmacol. Toxicol..

[155] Zeng W. (2020). Bisphenol A triggers the malignancy of nasopharyngeal carcinoma cells via activation of Wnt/β-catenin pathway. Toxicol. Vitr..

[156] Cowin P., Wysolmerski J. (2010). Molecular Mechanisms Guiding Embryonic Mammary Gland Development. Cold Spring Harb. Perspect. Biol..

[157] Wormsbaecher C., Hindman A.R., Avendano A., Cortes-Medina M., Jones C.E., Bushman A., Onua L., Kovalchin C.E., Murphy A.R., Helber H.L. (2020). In utero estrogenic endocrine disruption alters the stroma to increase extracellular matrix density and mammary gland stiffness. Breast Cancer Res..

[158] Speroni L., Voutilainen M., Mikkola M.L., Klager S.A., Schaeberle C.M., Sonnenschein C., Soto A.M. (2017). New insights into fetal mammary gland morphogenesis: Differential effects of natural and environmental estrogens. Sci. Rep..

[159] Soto A.M., Vandenberg L.N., Maffini M.V., Sonnenschein C. (2008). Does Breast Cancer Start in the Womb?. Basic Clin. Pharmacol. Toxicol..

[160] Soto A.M., Brisken C., Schaeberle C., Sonnenschein C. (2013). Does Cancer Start in the Womb? Altered Mammary Gland Development and Predisposition to Breast Cancer due to in Utero Exposure to Endocrine Disruptors. J. Mammary Gland Biol. Neoplasia.

[161] Tchen R., Tan Y., Barr D.B., Ryan P.B., Tran V., Li Z., Hu Y.-J., Smith A.K., Jones D.P., Dunlop A.L. (2022). Use of high-resolution metabolomics to assess the biological perturbations associated with maternal exposure to Bisphenol A and Bisphenol F among pregnant African American women. Environ. Int..

[162] Mandrup K., Boberg J., Isling L.K., Christiansen S., Hass U. (2016). Low-dose effects of bisphenol A on mammary gland development in rats. Andrology.

[163] Tharp A.P., Maffini M.V., Hunt P.A., VandeVoort C.A., Sonnenschein C., Soto A.M. (2012). Bisphenol A alters the development of the rhesus monkey mammary gland. Proc. Natl. Acad. Sci. USA.

[164] Fernandez S.V., Huang Y., Snider K.E., Zhou Y., Pogash T.J., Russo J. (2012). Expression and DNA methylation changes in human breast epithelial cells after bisphenol A exposure. Int. J. Oncol..

[165] Korsh J., Shen A., Aliano K., Davenport T. (2015). Polycyclic Aromatic Hydrocarbons and Breast Cancer: A Review of the Literature. Breast Care.

[166] Qin Q., Yang B., Liu Z., Xu L., Song E., Song Y. (2021). Polychlorinated biphenyl quinone induced the acquisition of cancer stem cells properties and epithelial-mesenchymal transition through Wnt/β-catenin. Chemosphere.

[167] Guo J.-Y., Wang M.-Z., Wang M.-S., Sun T., Wei F.-H., Yu X.-T., Wang C., Xu Y.-Y., Wang L. (2020). The Undervalued Effects of Polychlorinated Biphenyl Exposure on Breast Cancer. Clin. Breast Cancer.

[168] Parada H., Sun X., Tse C.-K., Engel L.S., Hoh E., Olshan A.F., Troester M.A. (2020). Plasma levels of polychlorinated biphenyls (PCBs) and breast cancer mortality: The Carolina Breast Cancer Study. Int. J. Hyg. Environ. Health.

[169] Schildroth S., Wise L.A., Wesselink A.K., Bethea T.N., Fruh V., Taylor K.W., Calafat A.M., Baird D.D., Henn B.C. (2022). Correlates of non-persistent endocrine disrupting chemical mixtures among reproductive-aged Black women in Detroit, Michigan. Chemosphere.

[170] Ahern T.P., Broe A., Lash T.L., Cronin-Fenton D.P., Ulrichsen S.P., Christiansen P.M., Cole B.F., Tamimi R.M., Sørensen H.T., Damkier P. (2019). Phthalate Exposure and Breast Cancer Incidence: A Danish Nationwide Cohort Study. J. Clin. Oncol. Off. J. Am. Soc. Clin. Oncol..

[171] Mao W., Jin H., Guo R., Chen P., Zhong S., Wu X. (2024). Distribution of parabens and 4-HB in human blood. Sci. Total Environ..

[172] Tapia J.L., McDonough J.C., Cauble E.L., Gonzalez C.G., Teteh D.K., Treviño L.S. (2023). Parabens Promote Protumorigenic Effects in Luminal Breast Cancer Cell Lines With Diverse Genetic Ancestry. J. Endocr. Soc..

[173] Hager E., Chen J., Zhao L. (2022). Minireview: Parabens Exposure and Breast Cancer. Int. J. Environ. Res. Public Health.

[174] Downs C.A., Amin M.M., Tabatabaeian M., Chavoshani A., Amjadi E., Afshari A., Kelishadi R. (2023). Parabens preferentially accumulate in metastatic breast tumors compared to benign breast tumors and the association of breast cancer risk factors with paraben accumulation. Environ. Adv..

[175] Darbre P.D., Aljarrah A., Miller W.R., Coldham N.G., Sauer M.J., Pope G.S. (2004). Concentrations of parabens in human breast tumours. J. Appl. Toxicol..

[176] Robin J., Binson G., Albouy M., Sauvaget A., Pierre-Eugène P., Migeot V., Dupuis A., Venisse N. (2022). Analytical method for the biomonitoring of bisphenols and parabens by liquid chromatography coupled to tandem mass spectrometry in human hair. Ecotoxicol. Environ. Saf..

[177] Park Y., Jang J., Park J., Kim J.H., Kim E., Song Y., Kwon H. (2014). Analysis of parabens in dentifrices and the oral cavity. Biomed. Chromatogr..

[178] Dualde P., Pardo O., Corpas-Burgos F., Kuligowski J., Gormaz M., Vento M., Pastor A., Yusà V. (2020). Biomonitoring of parabens in human milk and estimated daily intake for breastfed infants. Chemosphere.

[179] Park N.-Y., Cho Y.H., Choi K., Lee E.-H., Kim Y.J., Kim J.H., Kho Y. (2019). Parabens in breast milk and possible sources of exposure among lactating women in Korea. Environ. Pollut..

[180] Andersen M.H.G., Zuri G., Knudsen L.E., Mathiesen L. (2021). Placental transport of parabens studied using an ex-vivo human perfusion model. Placenta.

[181] Zhang H., Quan Q., Li X., Sun W., Zhu K., Wang X., Sun X., Zhan M., Xu W., Lu L. (2020). Occurrence of parabens and their metabolites in the paired urine and blood samples from Chinese university students: Implications on human exposure. Environ. Res..

[182] Zamora-León P. (2021). Are the Effects of DES Over? A Tragic Lesson from the Past. Int. J. Environ. Res. Public Health.

[183] Stillwater B.J., Bull A.C., Romagnolo D.F., Neumayer L.A., Donovan M.G., Selmin O.I. (2020). Bisphenols and Risk of Breast Cancer: A Narrative Review of the Impact of Diet and Bioactive Food Components. Front. Nutr..

[184] Dumitrascu M.C., Mares C., Petca R.-C., Sandru F., Popescu R.-I., Mehedintu C., Petca A. (2020). Carcinogenic effects of bisphenol A in breast and ovarian cancers (Review). Oncol. Lett..

[185] Yang P.-J., Hou M.-F., Ou-Yang F., Hsieh T.-H., Lee Y.-J., Tsai E.-M., Wang T.-N. (2022). Association between recurrent breast cancer and phthalate exposure modified by hormone receptors and body mass index. Sci. Rep..

[186] Cohn B.A., La Merrill M., Krigbaum N.Y., Yeh G., Park J.S., Zimmermann L., Cirillo P.M. (2015). DDT Exposure in Utero and Breast Cancer. J. Clin. Endocrinol. Metab..

